# Primary carcinoma of the fallopian tubes: Analysis of sixteen patients

**DOI:** 10.4274/tjod.67355

**Published:** 2015-06-15

**Authors:** Meryem Eken, Osman Temizkan, Ecmel Işık Kaygusuz, Dilşad Herkiloğlu, Ebru Çöğendez, Ateş Karateke

**Affiliations:** 1 Zeynep Kamil Training and Research Hospital, Clinic of Gynecology and Obstetrics, İstanbul, Turkey; 2 Şişli Hamidiye Etfal Training and Research Hospital, Clinic of Gynecology and Obstetrics, İstanbul, Turkey; 3 Zeynep Kamil Training and Research Hospital, Clinic of Pathology, İstanbul, Turkey

**Keywords:** Adnexial mass, primary fallopian tube carcinoma

## Abstract

**Objective:**

The aim of this study was to review patients with tubal carcinoma who underwent surgery in our clinic due to primary carcinoma of the fallopian tubes, a very rare gynecologic malignancy.

**Materials and Methods:**

Sixteen patients who were diagnosed as having primary carcinoma of the fallopian tubes and underwent surgery in Zeynep Kamil Research and Training Hospital between January 2007 and December 2014 were included in the study. Demographic data such as age, gravidity, parity, menopausal condition, symptoms, adjuvant therapy, recurrence of tumor, as well as time and type of operation were extracted from patient epicrisis reports and oncology files. Patient information was extracted from the patients’ current files and phone calls were made with patients and their relatives.

**Results:**

The mean age of patients was 59.6 (range, 43-78) years. Seventy-five percent of the women were menopausal at admission; the mean menopause duration was 10 years (range, 1-20 years). None of the patients were nulliparous and mean parity was 4.3 (2-8). The most common presenting symptom was abdominopelvic pain, followed by abnormal uterine bleeding. The most common histopathologic type was high-grade serous carcinoma. The mean follow-up duration was 23.7 months (range, 2-53 months). During follow-up, recurrence was seen in 4 (25%) patients. One patient left the study during follow-up. The mean disease-free survival was 48 months. No relation was found between disease-free survival, age, stage, grade, and histologic type in univariate logistic regression analysis.

**Conclusion:**

Primary carcinoma of the fallopian tubes is a rare gynecologic tumor that is seen in older patients, has no specific signs, and usually cannot be diagnosed before surgery. Therefore, we think that large-series, multi-centered studies with long-term follow-up duration are needed to define its etiopathogenesis and treatment strategies for the disease.

## INTRODUCTION

Primary fallopian tube carcinoma (PFTC) is a rarely-seen tumor that makes up approximately 0.14-0.18% of gynecologic malignancies^(1,2)^. Because PFTC has the same clinical findings and pathologic characteristics as serous epithelial ovarian carcinoma (EOC) and primary peritoneal serous carcinoma (PPSC) its actual incidence is lower, but some studies have indicated that its frequency is on the rise^(3,4)^.

The diagnostic criteria of PFTC have definite rules: The mass lesion in question must be located within the fallopian tube; the hystopathologic appearance of the tumor must reflect tubal epithelium; there should not be any pathologic finding of uterus and ovaries, or must include fewer tumors than the fallopian tube; and there must be a transition from benign to malignant epithelium^(5)^. In accordance with these criteria, PFTC is rare and there is always the possibility that some tubal lesions go unnoticed due to limitations in sampling.

It is stated that hormonal, reproductive, and possible genetic factors thought to increase the risk for EOC are also valid for PFTC^(6,7)^. On the other hand, in accordance with EOC treatment guidelines, PFTC is also treated through surgical staging, debulking, or adjuvant chemotherapy^(8,9)^. However, as PFTC is a rarely seen disorder, there is not enough treatment and follow-up experience with these tumors. Hence, its method of treatment and follow-up has yet to be defined in detail^(10,11)^.

Due to the rareness and adverse outcomes of PFTC, we believe that this issue must be rigorously scrutinized. Thus, our aim was to discuss patients whose tubal carcinoma was diagnosed and treated surgically in our clinic in light of relevant literature.

## MATERIALS AND METHODS

Sixteen patients who were diagnosed as having PFTC in line with the diagnosis criteria of Sedlis et al. and underwent surgery at the Zeynep Kamil Training and Research Hospital Oncology Clinic between January 2007, and November 2014, were included in the study^(5)^. Ethical approval was obtained from the institutional ethics committee. Informed consent was obtained from each patient. Factors such as age, gravidity and parity, menopausal condition, symproms reported by the patient on presentation, adjuvant therapy, tumor recurrence, and previous surgical procedures were obtained from the patients’ oncology reports. Information about follow-up was collected from the the patients’ current files or by contact through the patients’ relatives or close acquaintances. Patients who were diagnosed as having PFCT but did not come for their follow-ups or whose follow-up information could not be obtained were excluded from the study.

Relative to operation information and pathology results, the patients were categorized in accordance with the staging system of the International Federation of Gynecology and Obstetrics (FIGO)-2014^(12)^. Hystologic grading was performed on the basis of the structural characteristics of the tumor. Grade 1 refers to poorly-differentiated, Grade 2 to moderately differentiated, and Grade 3 to well-differentiated tumors. Full surgical resection was defined as patients who had no residual tumor larger than 1 cm in size.

Surgery was selected as the primary treatment for all patients and each received chemotherapy following surgery. As a chemotherapy procedure, they received a total of 6 cycles in every three weeks, consisting of Paclitaxel (175 mg/m2) and platinum-based chemotherapy (Carboplatin (6 mg/mL)).

Statistical analysis was performed by SPSS 16 software. Chi-Square test was used for categorical variables. T-test was used for normally distributed variables. And for non-normally distributed variables, Mann-Whitney U-test was used. Disease free survival rate was calculated on the basis of the Kaplan-Meier curve. As regards analysing survivalwithout disease on the basis of categorical variables, log-rank test was used for univariate analsis and a comparison was thus made. The relation between the levels of CA-125 and factors such as the age of patients, mass diameter and tumor stage was evaluated through multivariate analysis. If p value was below 0.05, it was accepted as statistically significant.

## RESULTS

In total, 93 patients underwent surgery in our clinic due to ovarian cancer between January 2007, and November 2014. Among these patients, 83 were diagnosed as having high-grade serous carsinoma (HGSCs). Sixteen of these 83 (19.2%) were reported as PFTC.

As seen in Table 1, the mean age of patients was 59.6 years (range, 43-78 years). During the application process, 12 of the patients (75%) were in menopause and the mean menopause period was 10 years (range, 1-20 years). None of the patients were nulliparous and mean parity was 4.3 (min 2, max 8).

The presenting symptoms of the patients were: abdominal pain in 12 patients (75%), 3 (18.7%) had postmenopausal bleeding, 3 (18.7%) had menorrhagia, and 1 (6.3%) had supraclavicular lymphanedopathy. Endometrial sampling of the three patients with postmenopausal bleeding revealed an endometrial polyp in one patient, and findings of atrophy in two patients. The pathologic results of the patients with menorrhagia revealed an endometrial polyp in two patients and proliferative endometrium in one.

None of the patients’ tumors were diagnosed preoperatively. Twelve (75%) patients underwent surgery due to an adnexal mass after an initial diagnosis of ovarian cancer, 3 had surgical procedures (18.7%) due to benign reasons (hydrosalpinx, menometrorrhagia, postmenopausal bleeding), and 1 (6.3%) due to peritonitis carcinomatosa.

With respect to postoperative hystopathologic evaluation, 14 patients (87.5%) were diagnosed as having high-grade serous adenocarcinoma and 2 patients (12.5%) had mixed tumors (Müllerian carcinoma in one patient and undifferentiated carcoma component in the other).

The median preoperative CA-125 value was 292 U/mL (min 3.8-max 6713). In the preoperative period, one patient’s CA-125 value was not measured.

Three patients’ (18.7%) CA-125 values were within normal limits; two of these patients had a low-grade tumor and the other’s was high-grade. Additionally, an evaluation of the stage of patients with normal CA-125 values revealed that one was in the advanced stage (Stage 3c) and the other two were in (Stage 1a2/2a). The multivariate analysis of the relation between CA-125 values before surgery and factors such as the age of patients, mass diameter, and tumor stage did not indicate any significant relation (p=0.35).

In the surgical staging, similar to EOC treatment, we performed abdominal washing cytology, total hysterectomy and salpingo-oophorectomy, pelvic/paraaortic lymph node dissection, omentectomy, excisional biopsy from suspected lesions, and appendectomy (if indicated). In our study, full surgical staging was achieved in 14 patients (87.5%) during the operation; diagnosis was made on the basis of the pathology result in the other two (12.5%) patients; therefore full surgical staging was completed with second look laparatomy. Eight of the 16 patients underwent appendectomy: 2 were at early stages (Stage 1A2 and Stage 2B) and the others were at advanced stages (Stage 3-4). Moreover, full surgical resection could not be achieved in two (12.5%) patients during surgery because the tumors were at an advanced stage. In summary, 3 patiens were classified as Stage 1, 2 patients as Stage 2, 10 patients as Stage 3, and 1 patient as Stage 4.

**Figure 1:**
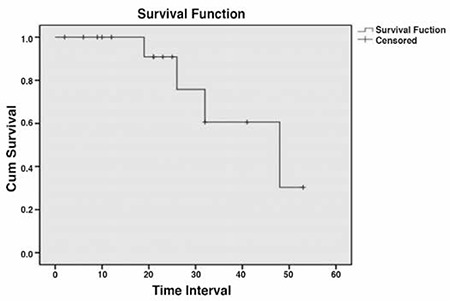
Analyses of disease free survival patients with primary fallopian tube carcinoma

The median follow-up duration was 23.7 months (range, 2-53 months). During follow-up, 4 of the 16 patients (25%) relapsed. One patient left the study dring follow-up (total duration of survival was 53 months). The median disease-free period was 48 months (Figure 1). The characteristics of patients who relapsed are summarized in Table 2.

All patients received a postoperative chemotherapy protocol consisting of 6-8 cures of paclitaxel and carboplatin. No patient received any radiotherapy. Clinico-pathologic factors related with the disease-free survival rate are presented in Table 3.

## DISCUSSION

Ovarian, tubal, and ‘primary’ carcinomas, which are defined as high-grade serous carcinomas (HGSCs) in the context of female genital cancers, attract the attention of clinicians and researchers because they are often at an advanced stage, rapidly spread, 3 nd are hard to diagnose.

The pathogenesis of HGSC, which stem from ovarian surface epithelium, is not known^(13)^. Recent observations indicate that the majority of serous tubal intraepithelial carcinomas (STIC) can be a precursor lesion for fallopian tube, ovarian, and peritoneal HGSCs^(14,15)^.

STICs are lesions limited to the epithelium of the fallopian tube. It was first observed in the distal fallopian tubes (fimbriae) that were prophylactically obtained from women at high risk of developing ovarian cancer due to BRCA mutations^(16)^. Approximately 10-15% of patients who were diagnosed as having fallopian tube (STIC lesions) were identified in this manner^(17)^. Moreover, STIC was observed among 50-60% of cases of sporadic pelvic HGSC. Among 83 cases of pelvic HGSC (tubal-peritoneal-ovarian) that were surgically treated in our clinic between 2007 and 2014, the incidence of PFTC was 19.2% (n=16).

PFTC is most often observed in the fourth decade of life; the median age of incidence is 64 years^(18)^. In our study, the mean age of patients was 59.6 years (range, 43-78 years). During the diagnosis process, 12 of the patients (75%) were in menopause and the mean menopause period was 10 years (range, 1-20 years). These findings are in agreement with the relevant literature^(19)^.

Fallopian tube carcinoma has heterogeneous clinical findings. The most frequent symptoms and findings are abdominal pain, which might be colic as a result of the narrowed tubal peristalsis or increased tubal distension, vaginal bleeding, and watery discharge. A previous study reported Latzko’s triad of intermittent and watery serosanguineous vaginal discharge, colicky pain that often regressed with discharge, and a pelvic mass in 15% of patients^(3)^. In concert with the relevant literature, the patients included in our study most frequently presented with abdominal pain (75%). The second most frequent symptom was abnormal uterine bleeding (menorrhagia or postmenopausal bleeding). Scholz et al. first reported a patient who presented with a supraclavicular mass, and later Sakurai et al.^(20,21)^. In our study, there was also a patient with a swelling in her supraclavicular lymph node. A fine-needle aspiration biopsy revealed an adenocarcinoma. Afterwards, positron-emission tomography (PET-CT) imaging was performed in order to determine the primary focus. This indicated a mass at the left adnexal site. The patient was later diagnosed as having PFTC and thus underwent surgery.

PFTC’s metastasis pattern is similiar to that of ovarian cancer and the most frequent are intraperitoneal metastases. PFTC’s nodal spread gravitates towards retroperitoneal lymph nodes because fallopian tubes are rich in lymphatic vessels, which provide drainage for paraaortic lymph nodes through infudibulopelvic lymphatics^(7)^. Lymph node metastases have been reported in 33% of cases with different PFTC stages^(22)^. Nonetheless, PFTC rarely gives rise to a metastasis in supraclavicular lymph nodes.

It is clear that there is a strong genetic susceptibility for breast and tubal cancer and it seems that BRCA1 and -2 mutations are important risk factors for tubal carcinoma^(23)^. Slanez et al. reported that its rate of co-existence with breast cancer is 35%. In our study, only one patient had a history of breast cancer^(24)^.

No patients’ disease was preoperatively diagnosed in our study. All patients who had preoperative imaging were underwent surgery after being initially diagnosed as possibly having an ovarian carcinoma. Three (18.7%) patients’ PFTC was incidentally diagnosed. One patient was being followed-up due to breast cancer and underwent surgery due to menorrhagia, another patient underwent surgery due to hydrosalpinx and was diagnosed as having PFCT after the postoperative pathology result. The remaining patient underwent surgery after being diagnosed as having an endometrial polyp due to postmenopausal bleeding, but ovarian cancer was diagnosed during the operation.

PFCT has a low rate of preoperative or intraoperative description^(7)^. The rate of preoperative description varies between 0-10%^(25,26)^.

It is challenging to diagnosis PFTC radiologically and the majority of patients are preoperatively diagnosed as having ovarian carcinoma^(27)^. Ultrasonography is the fundamental imaging for the diagnosis of adnexal masses. In ultrasonography, papillary projections with anechoic contents and a decreased echogenicity or intraluminal masses point to PFTC^(28)^. However, the majority of fallopian tubes have a non-specific ultrasonographic imaging that imitates other pelvic diseases such as tubo-ovarian abscess, ovarian tumors, and ectopic pregnancy^(7)^. In the preoperative images made within our study, no specific findings suggested tubal cancer, and all patients with a preoperative imaging underwent surgery after receiving an initial diagnosis of ovarian carcinoma.

The most frequent hystologic type is serous carcinoma. Its microscopic appearance is almost the same as that of serous ovarian carcinoma. Its less frequent variants are endometrioid-, clear-celled-, transitional-celled-, and undifferentiated carcinoma^(29)^. In previous studies, 80.6% of patients were reported to have a poorly-differentiated carcinoma^(30,31)^. In our study, the most frequent hystopathologic finding was high-grade serous carcinoma.

Many tumor markers have been reported to be capable of increasing preoperative diagnostic accuracy and contribute to postoperative follow-up. CA-125 is the one of the most-frequently used and it is often high in cases of PTFC. CA-125 serum levels were reported to be increased before the treatment in 80% of patients in one study^(27)^. Hefler et al. reported that the mean preoperative CA-125 levels of patients with PFTC was 183 U/mL and stated that during their follow-up, the sensitivity, specificty, and positive and negative predictive values of serum CA-125 levels of were, 92%, 90%, 67%, and 98%, respectively. In our study, the mean CA-125 levels of patients and high preoperative rate of incidence of CA-125 were similar to those in other studies^(32)^. Moreover, no significant relation was found between the CA-125 levels in PFTC before surgery and factors such as age, mass diameter, and tumor stage.

Similar to the surgical treatment of EOC, the aim in the treatment of PFTC is the complete removal of the tumor^(33)^. The standard surgical treatment is total abdominal hysterectomy, bilateral salpingo-oophorectomy, appendectomy, infracolic omentectomy, pelvic and paraaortic lymph node dissection, abdominal fluid washing, and peritoneal sampling^(34)^. Klein et al. stated that the five-year survival rate of total hysterectomy and bilateral salpingooophorectomy was only 58% and that full resection, including additional radical lymphadenectomy, provided for a better rate of survival (33%)^(35)^.

There are studies that suggest that appendectomy should be routinely performed in surgical staging or that appendectomy should not be performed in early stages. In Ayhan et al.’s study, which comprised 285 cases of epithelial ovarian cancer, metastases to the appendix were found at a rate of 37%. Some 4.9% of these cases were early-stage, but the stage was advanced due to an isolated metastasis to the appendix. Consequently, the authors recommended routine appendectomy in surgical staging^(36)^.

Conversely, Ramirez et al. did not recommend routine appendectomy, even though no increase was observed with respect to complications in early-stage ovarian cancers^(37)^.

In the present study, appendectomy was performed for 8 patients in total; 2 were at an early-stage (Stage 1A and 2B), and the others were advanced stage (Stage 3-4).

For patients at an advanced stage, it is necessary to remove as many tumor masses as possible and to perform aggressive cytoreductive surgery^(38)^. In our study, full surgical resection was achieved in 14 patients; full surgical resection was not possible in 2 patients because their disease was at an advanced stage. These two patients relapsed.

For over fifteen years, the preferred treatment protocol in PFTC has been a combination of taxane and platinum-based chemotherapy^(39)^. In our study, all patients received 6 cures of adjuvant paclitaxel and carboplatin chemotherapy, regardless of the post-surgical stage.

FIGO staging is the most coherent prognostic factor related with survival^(40,41)^. Yu et al. reported that the five-year survival rate of 64 patients with PFTC was 56.3%^(42)^. Other researchers found that the three- and five-year survial rate of PFTC patients were 87.3% and 65.2%, respectively^(16)^. Ma et al. stated that the three- and five-year survival rate of patients with PFTC were 80.7% and 65.4%, respectively^(43)^. As a result of multiple factor analysis, they showed that there was a correlation between an intraoperative residual tumor diameter >1 cm and a prognosis of metastasis to the omentum. Naniah et al. reported that the mean duration of survival without progression was 19 months (range, 15-21 months) and the mean overall survival duration was 27 months (range, 22-36 months)^(6)^. The mean follow-up duration in our study was 23.7 months (range, 2-53 months). The mean disease-free period was 48 months and 4 patients (25%) relapsed during follow-up.

In the regression analysis that evaluated factors that had an impact upon survival without any disease, it was observed that age, stage, grade or hystologic type had no effect on survival without disease.

The most important prognostic factors for survival were reported to be stage, patient’s age, and residual tumor after the first surgery in patients with advanced-stage disease^(44,45)^.

Survival analysis could not be done in our study because the number of patients was too small and no patient was followed-up for at least 2 years. Moreover, no multivariate analysis could be done for prognostic factors because there were too few patients. These were the main shortcomings of our study.

## CONCLUSION

PFTC are poorly-differentiated serous cancers that can lead to non-specific symptoms and as such, a correct diagnosis can often be missed. It is thought that it has a strong relation with breast cancer and its treatment and follow-up are usually planned according to EOC guidelines. The low incidence of tumor and the usual difficulty in making a diagnosis prior to surgery considerably decrease the opportunity to undertake randomized research about patients with fallopian tube cancer. Therefore, large-series, multi-center studies with long follow-up periods will be important in determining the etiopathogenesis and treatment strategy of the disorder.

## Figures and Tables

**Table 1:**
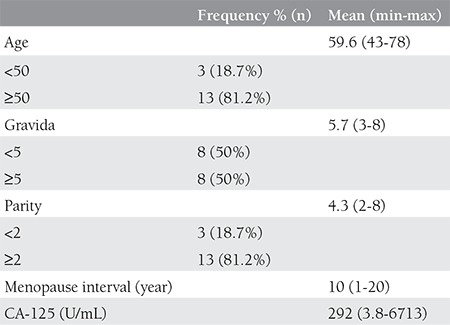
Patients clinical and demographic characteristics

**Table 2:**
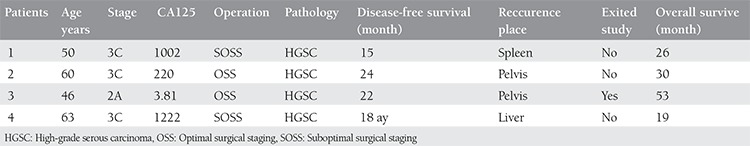
Characteristics of patients with recurrent disease

**Table 3:**
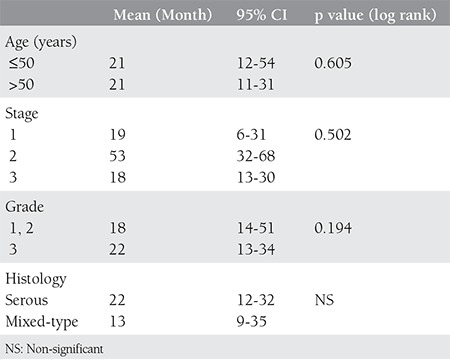
Comparisons of univariate analyses for disease-free survival of the patients
